# Factors associated with data quality in the routine health information system of Benin

**DOI:** 10.1186/2049-3258-72-25

**Published:** 2014-07-28

**Authors:** Yolaine Glèlè Ahanhanzo, Laurent T Ouedraogo, Alphonse Kpozèhouen, Yves Coppieters, Michel Makoutodé, Michèle Wilmet-Dramaix

**Affiliations:** 1 Epidemiology and Biostatistics Department, Public Health Regional Institute, University of Abomey-Calavi, Abomey-Calavi, Benin; 2 Center of research in Epidemiology, Biostatistics and Clinical Research, School of Public Health, Université Libre de Bruxelles, Bruxelles, Belgium; 3 Environment and Health Department, Public Health Regional Institute, University of Abomey-Calavi, Abomey-Calavi, Benin

**Keywords:** Data quality, Related factors, Health information systems

## Abstract

**Background:**

Routine health information systems (RHIS) are crucial to the acquisition of data for health sector planning. In developing countries, the insufficient quality of the data produced by these systems limits their usefulness in regards to decision-making. The aim of this study was to identify the factors associated with poor data quality in the RHIS in Benin.

**Methods:**

This cross-sectional descriptive and analytical study included health workers who were responsible for data collection in public and private health centers. The technique and tools used were an interview with a self-administered questionnaire. The dependent variable was the quality of the data. The independent variables were socio-demographic and work-related characteristics, personal and work-related resources, and the perception of the technical factors. The quality of the data was assessed using the Lot Quality Assurance Sampling method. We used survival analysis with univariate proportional hazards (PH) Cox models to derive hazards ratios (HR) and their 95% confidence intervals (95% CI). Focus group data were evaluated with a content analysis.

**Results:**

A significant link was found between data quality and level of responsibility (p = 0.011), sector of employment (p = 0.007), RHIS training (p = 0.026), level of work engagement (p < 0.001), and the level of perceived self-efficacy (p = 0.03). The focus groups confirmed a positive relationship with organizational factors such as the availability of resources, supervision, and the perceived complexity of the technical factors.

**Conclusion:**

This exploratory study identified several factors associated with the quality of the data in the RHIS in Benin. The results could provide strategic decision support in improving the system’s performance.

## Background

Routine Health Information Systems (RHIS) contain information derived at regular intervals from mechanisms designed to meet predictive information needs. As decision support tools, these systems are crucial to health system planning. RHIS performance is judged on the production of quality information and its use in decision-making. Statistics Canada defines the quality of its statistical information according *to the data*’*s fitness for use by* customers and applies six quality criteria: relevance, accessibility, validity, coherence, timeliness and interpretability [1]. In the development of the Performance of Routine Information Systems (PRISM) concept, John Snow Inc. and Measure defined information quality according to the following characteristics: coverage (sector, region, district, etc.), accuracy, comprehensiveness/timeliness, collection frequency, and information communication process [2-4]. Quality information, therefore, is information that is comprehensive, accurate, specific and useful. As information is the product of data transformation, the quality of the information in the RHIS means quality data, which equates to the data’s comprehensiveness, validity, accuracy, and fitness for use, among other benefits.

Although operational in most developing countries, the RHIS in those countries are described as ineffective for several reasons: insufficient comprehensiveness of the information, the poor quality of the data collected, and low levels of use in real-time decision-making. Traditionally, according to the literature, the critical factors in RHIS performance are environmental and organizational (availability of resources), technical (complexity of the collection tools in their format and procedures for use and complexity of the technologies used) and behavioral (staff motivation and competence associated with RHIS tasks) [4].

The RHIS in Benin, called the “Système National d’Information et de Gestion Sanitaire” (SNIGS), has been the subject of various evaluations, which have found the quality of the data to be poor [5-7]. The reasons for these findings are insufficient resources for training staff and implementing support activities such as supervision as well as low staff motivation.

To help improve the performance of the RHIS in Benin, the objective of this study was to identify the factors associated with poor data quality, especially those linked to environmental, organizational, technical and behavioral human resources.

## Methods

### Type of study

This was a cross-sectional, descriptive and analytical study conducted between October and November 2012.

#### Context, study population and ethical considerations

In Benin, the RHIS is organized according to the pyramid structure of the healthcare system and includes the public and private sectors. The illegal facilities of the private sector are not included. Furthermore, the resources available for training and supervision are mostly dedicated to the public sector. As in most developing countries, the RHIS is composed of data collected from patient’s information records. These data are assembled in periodic summary reports produced by health staff in outlying areas. These reports are activity summary tables that are filled in using the data from the records. The reporting forms are linked to outpatient care, maternal care, immunization, financial management, laboratory, and anti-malarial activities. The number of data items is variable depending on the form. The shortest is the anti-malarial activities reporting form with 45 items; the longest is the outpatient care reporting form with 815 items. There is no computer in the health facilities; thus, the reporting forms are completed by hand. One person, often the facility manager, is charged with completing the reporting forms. When there are enough human resources in the same health facility, the head of the health district can assign one person to each type of reporting form. Accordingly, in the same facility, one or more persons are designated to complete the different reports, but the same person completes one type of reporting form. No data are collected about the time taken to complete the different reporting forms. The reporting forms are filled in monthly and are periodically sent, typically monthly, to the district health management team. The facility manager must analyze the report and use the information for decision making; however, information use is still insufficient. Computerization (still rudimentary) is usually performed at the health district level. A Microsoft Access database is created and sent to the intermediate level, and then to the central level.

This study was conducted in first-line public and private health centers of the RHIS in four municipalities of the department of Atlantique-Littoral in the south of Benin. The municipality of Cotonou was selected deliberately because it is the only urban municipality in the department. Three rural municipalities were selected randomly from among the department’s eight rural municipalities. All of the public and private health centers in the three rural municipalities were selected. In the city, where the workforce in the private health centers was very large and unmanaged, random sampling was performed from the list available in order to select equal numbers of private and public health centers. The population targeted by the study were health workers responsible for RHIS data in the health centers. We also collected all of the data generated by each health worker in the twelve months preceding the survey. Sixty-nine public and private health centers including one hundred and twenty health workers were included in the study.

The research protocol for this study was approved by the Benin National Ethics Committee for Research in Health (Comité National d’Ethique pour la Recherche en Santé) and also by the regional authorities and the local health authorities. The participants were informed about the objectives and anonymity of the survey. They were invited to give their informed consent before receiving the questionnaire.

### Variables, techniques and collection tools

The dependent variable was the quality of the data generated by the health worker (good versus poor). The independent variables were:

•socio-demographic characteristics: age, gender, general level of education, basic vocational training;

•work-related characteristics: work location (urban/rural), sector of employment (public/private), type of contract (open-ended/fixed-term), responsibility of the health center (Yes/No), RHIS training or retraining in the last 12 months (Yes/No), supervision concerning RHIS data quality received in the last six months, receipt of financial incentives and availability of material resources for RHIS activities;

•the perceived complexity of the technical factors (Yes/No),

•the personal resources of the health worker such as their work engagement and their perceived self-efficacy concerning RHIS activities.

The techniques and tools used included a document review with a processing form to assess the quality of the data and an interview with a self-administered questionnaire to collect other information.

We also held two focus groups with health workers to determine the reasons for the poor quality of the data they produced. Health workers were selected for focus groups among all the health workers in charge of data collection in the municipalities involved in the study. They were randomly selected among the volunteers who wanted to participate in the focus group. Each group was composed of six people.

The quality of the data batch was assessed using the Lot Quality Assurance sampling (LQAS) method with n = 32 and d* + *1* = 3 (n: size of the sample and d* + 1: maximum number of defective units expected per sample). Other parameters included in the equation were: N large, P_0_ = 20%, P_a_ = 5%, N being the size of the batch, P_0_ proportion of defective units, and P_a_ the maximum proportion of defective units expected to consider the lot of good quality [8-10]. The batch was defined as all of the data generated by a single health worker in the twelve months preceding the survey. The data were all numerical values that had to be entered in the periodic report produced by the health worker. The quality of the data randomly sampled, was assessed for comprehensiveness, reliability and accuracy. In this study, data comprehensiveness was defined as the “availability of the data across all of the documents in which it must be provided” for the twelve months. If the data were missing or if the document had not been produced by the health worker, the data were considered to be incomplete. In this study, data reliability was defined as “case correspondence to the case definition in the national guidelines”. The data were judged as being unreliable if the cases reported did not correspond with the case definition. Verification of reliability in this study was based on the clinical information entered in the records and did not involve verification with the actual clinic concerning the patient. For example, if the reporting form mentioned 5 cases for simple malaria, the surveyor checked every case reported in the register source: Does each case correspond to “fever + positive rapid diagnostic test” as recommended in the national guidelines? If one reported case did not match the national definition used in the guidelines, we considered the data to be unreliable. Data accuracy was defined by “the numerical correspondence between the data recorded in the document and that in the record”. A relative difference of 5% was permitted. Data were judged as lacking if they did not meet all three criteria. The batch was rejected if three defective items of data were found in 32 random samples. In each batch, 32 data items are expected to be randomly sampled with tables of random numbers. The sampling was stopped as soon as the maximum number of 3 defective items of data was reached. The number of samples prior to the batch’s rejection was, therefore, variable. The quality of the data generated by a health worker was judged as poor if his/her data batch was rejected.

Work engagement for RHIS activities was measured using the French version of the Utrecht Work Engagement Scale (UWES) with 9 items [11-14] and was adapted to the field of health information system activities. The UWES comprised 3 items for each dimension: absorption, dedication and vigor. Each item was rated on the Likert scale from 0 (situation never found) to 6 (situation found every day). An average score was obtained for each dimension and for the overall scale (ranging between 0 and 6). A high score reflected a high level of engagement by the health worker in RHIS activities. In the analyses, the overall score for work engagement was divided into two categories obtained by applying the median threshold (high level of engagement versus low level of engagement).

Perceived self-efficacy was measured with the HMIS tasks self-efficacy questionnaire (confidence level in their own abilities) taken from the Organizational and Behavioral Assessment Tool (OBAT) used to assess the Performance of Routine Health Information Systems (PRISM) in contexts similar to that of Benin [15,16]. In seven questions, the subject was invited to rate his/her perceived self-efficacy in performing various RHIS tasks on a scale of 0 to 100%. The average score obtained for the seven questions expressed as a percentage was used. A high score reflected a high level of perceived self-efficacy and a high level of confidence in the health worker’s own abilities. This score was recoded based on a median threshold in two categories of perceived self-efficacy: High and Low.

### Analysis

The descriptive statistics used were the average and its standard deviation for the scores and percentages for the qualitative variables. The percentages were compared using the chi^2^ test. For the analytical component, we conducted survival analysis using the rejection of the data batch as the event and the number of samples before the rejection as the time variable. Univariate proportional hazards (PH) Cox models were applied to derive hazard ratios (HR) and their 95% confidence intervals (95% CI) and p-values using the Wald test. The proportional hazard assumption was checked using the test and plots based on Schoenfeld residuals and examining the plots ln (-ln (S (t)); S (t) is the survival curve. We presented Kaplan Meier survival curves to compare the probability of batch rejection (probability that the data batch would be of poor quality) according to the independent variables. The median number of samples (and interquartile range) was presented. We also performed a comparison of the average scores for work engagement and perceived self-efficacy based on the quality of the batches using the Student t-test. The significance threshold used was 5%.

For the qualitative component concerning the focus group, information was categorized by topic, and a content analysis was completed.

## Results

### Description of the sample

Among the 120 health workers asked to participate in the survey, 116 accepted, yielding a non-response rate of 3.3%. The sample was composed of 69.3% women. The majority of the participants were healthcare staff, nurses or midwives (81.7%); 17.8% of the participants had higher education backgrounds. The majority of the participants were working in the public sector (74.8%), and less than a quarter (22.4%) had been trained or retrained in the RHIS in the last 12 months. The average age was 41.2 ± 8.5 years in the public sector and 39.5 ± 8.7 years in the private sector, and no significant difference were found between the 2 groups (p = 0.370). More women were from the public sector than the private sector (p = 0.024). Supervision on data quality, availability of material resources for RHIS and financial incentives for RHIS activities were not comparable among the public and private sectors (p = 0.000, p = 0.009 and p = 0.005, respectively) (Table 1). Among the health workers interviewed, 38.5% were also responsible for the health center in addition to their RHIS activities. Regarding the perceived complexity of the technical factors of the RHIS, 58.9% of the participants thought that the documents or procedures were complex. Regarding work engagement in RHIS activities, the average score was 4.1 (±1.5). The average score for perceived self-efficacy was 61.4% (±28.2). The quality of the data was deemed poor for114 out of 116 batches of health workers; 98.3% of the batches evaluated were rejected. For the comprehensiveness criteria, 103 of the 116 batches (88.8%) were rejected. For reliability and accuracy, 8.6% and 9.5% of the batches were rejected, respectively.

**Table 1:** **Gender and work related characteristics of health workers responsible for RHIS according to sector of employment**, **Benin 2012**

**Variables**	**Public facilities**		**Private facilities**		**P**
	**n**	%	**n**	%	
Gender	80		27		0.024
Female	60	75.0	14	51.9	
Work location	83		28		0.056
Urban	39	47.0	19	67.7	
RHIS training in the last 12 months	28		83		0.187
Yes	4	14.3	22	26.5	
Supervision on data quality	83		28		0.000
Yes	51	61.5	6	21.4	
Availability of material resources for RHIS activities	81		27		0.009
Yes	22	27.2	1	3.7	
Financial incentives for RHIS activities	79		26		0.005*
Yes	18	22.8	0	0.0	

*Fischer exact test.

### Univariate analysis

**Figure 1:**
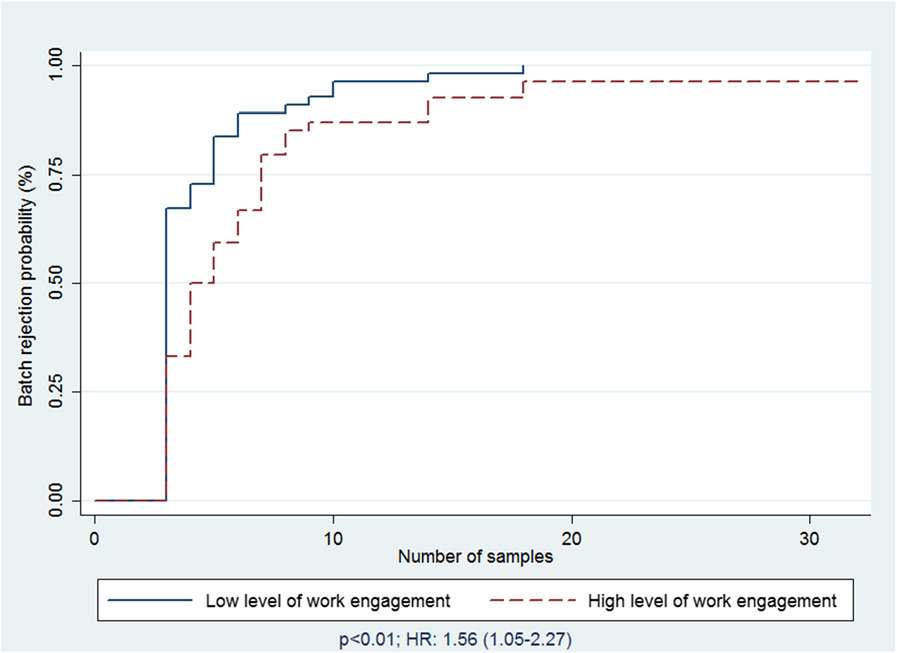
Batch rejection probability according to number of draws by level of work engagement.

**Figure 2:**
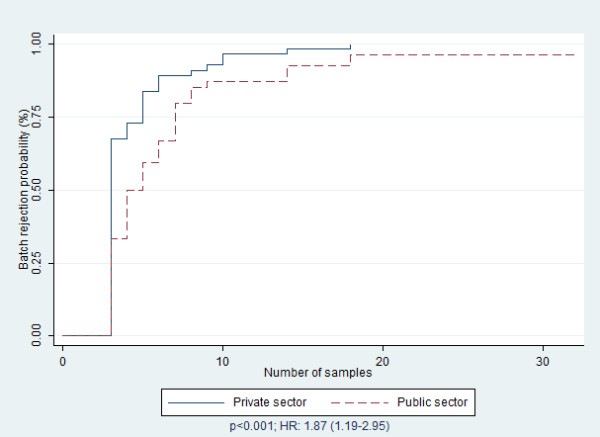
Batch rejection probability according to number of draws by sector of employment.

These findings are summarized in Table 2. The probability of batch rejection was lower among health workers who, in addition to their RHIS activities, were responsible for the health center; the health workers who were not responsible for the health center were significantly more at risk of rejection of their batch (HR: 1.52; p = 0.011). Likewise, the RHIS training or retraining in the last 12 months was a risk factor of batch rejection. Indeed, people who were not trained or retrained in RHIS in the previous 12 months were significantly more at risk of rejection of their batch compared to health workers who received training in RHIS in the previous 12 months (HR: 1.49; p = 0.026) (Table 2). This difference was also noted according to the level of the health worker’s engagement in RHIS activities (HR: 1.56; p < 0.01) (Figure 1) and the sector of employment (HR: 1.87; p < 0.001) (Figure 2). Therefore, a batch from the private sector is more at risk of rejection than a batch from the public sector, and a batch from people with a low level of work engagement is more at risk of rejection than a batch from a health worker with a high level of work engagement. There were no differences if the health workers received a financial incentive (p = 0.690), had the material resources (p = 0.078) or were supervised over the last six months (0.349). Likewise, the perception of the complexity of the technical factors was not significantly associated with the probability of the batch’s rejection (p = 0.466). This comparison, according to the two categories of perceived self-efficacy, was not statistically significant (p = 0.052).

**Table 2:** **Number of samples prior to batch rejection**, **Hazard Ratio** (**HR**) **and 95**% **confidence interval** (**CI**) **according to independent variables**

**Variables**	**n**	**Median number of samples prior to batch rejection**	**P25**-**P75**	**HR**	**95% CI**	**P**
Gender						
Female	73	4.0	(3–6)	1.47	0.96-2.22	0.079
Male	33	3.0	(3–4)	1		
Health center responsibility						
Yes	38	5.0	(3–8)	1.52	1.01-2.30	0.011
No	64	3.0	(3–5)	1		
General level of education						
University	16	5.0	(3–8)	1.47	0.86-2.50	0.165
Primary or secondary school	90	3.0	(3–5)	1		
Basic vocational training						
Others	20	3.0	(3–7)	1.15	0.70-1.85	0.593
Health care staff	87	3.0	(3–6)	1		
Work location						
Urban	57	3.0	(3–6)	0.99	0.69-1.44	0.971
Rural	52	3.0	(3–6)	1		
Sector of employment						
Private	28	3.0	(3–3)	1.87	1.19-2.95	0.007
Public	81	4.0	(3–7)	1		
Training or retraining on RHIS in the last 12 months						
Yes	26	6.0	(3–8)	1.49	1.06-2.38	**0.026**
No	90	3.0	(3–5)	1		
Availability of material resources						
Yes	22	4.0	(3–6)	0.89	0.56-1.43	0.078
No	84	3.0	(3–6)	1		
Supervision on data quality						
Yes	57	4.0	(3–6)	1.19	0.82-1.72	0.349
No	59	3.0	(3–5)	1		
Financial incentives						
No	87	3.0	(3–6)	0.89	0.53-1.51	0.690
Yes	18	3.0	(3–6)	1		
Perceived complexity of the technical factors						
No	43	4.0	(3–6)	1.15	0.78-1.71	0.466
Yes	62	3.0	(5–6)	1		
Perceived self efficacy						
Low	26	3.0	(3–4)	1.42	0.84-2.27	0.052
High	63	4.0	(3–6)	1		
Level of work engagement						
Low	55	3.0	(3–5)	1.56	1.05-2.27	<0.001
High	52	4.0	(3–7)	1		

As concerns the data quality judged on comprehensiveness which was the most important criteria of rejection, the average score for perceived self-efficacy varied significantly according to the quality of the batch. Indeed, the average self-efficacy score was higher for health workers whose batches had not been rejected based on comprehensiveness criteria (p = 0.03). The average score for work engagement was 4.2 within the group of health workers whose batches were not rejected, versus 3.4 in the group whose batches were rejected (p = 0.06) (Table 3).

**Table 3:** Averages scores of perceived efficacy and work engagement according to batches quality judged on comprehensiveness

**Variables**	**Mean (SD)**		**p**
	**Rejected batches (n** = **96)**	**Non rejected batches (n** = **13)**	
Perceived self efficacy (%)	60.0 (29.3)	72.0 (13.3)	0.03
Level of work engagement	3.4 (1.7)	4.2 (1.5)	0.06

### Results of the focus groups

According to the health workers, the main reasons for the poor comprehensiveness of the data were: the excessively high number of cells to complete in the documents; the restricted size of the cells; and the irrelevance of some data in relation to the services provided and their technical platform. According to one health worker: “*There are too many boxes to fill in and we waste a lot of time*, *especially in reporting zeros*”. Another health worker added: “*The boxes are so small that it is difficult to see and when we fill them in everything is illegible*.” The lack of accuracy and reliability could be linked to counting and reporting errors due to carelessness, the lack of motivation and engagement of some health workers in the activities, and interference by other tasks. Regarding the lack of accuracy and reliability, one health worker said: “*When you are a manager and you are in the middle of creating the report*, *you get called away for something else*, *and when you come back you make more mistakes or you are very busy with other activities and you do not check the report before sending it*”. One health worker added: “*When you are a manager*, *you take care to avoid errors in your report because you do not want the medical officer to criticize your work in front of your colleagues at the monthly meeting*”. The health workers did not appear to frequently use the guidelines as these were not up to date and also because using the guidelines would take time. The health workers also seemed to be insufficiently qualified, as few had been trained on RHIS and the training received had not always been appropriate. Some of their comments supported these findings: “*We are trained in one day in 6 or 7 tools concerning the clinic*, *vaccination*, *maternity*, *financial data*, *etc*”. “*Treatment protocols often change and old guidelines are not reviewed*, *so we do not always use them*”.

## Discussion

The aim of this study was to identify some factors associated with poor data quality in a system in which data acquisition is still performed manually. According to our results, the prevalence of data of poor quality is high. One reason for this phenomenon might be the nature of the criteria that we used, particularly the inclusion of the comprehensiveness of the data, as there is a major issue concerning the lack of comprehensiveness of the data in the reports. This issue was conveyed in the comments of the health workers, the outcomes of the focus groups, and several previous studies [15,17]. The work of Hotchiss et al. in Uganda concerning the assessment of the performance of the RHIS reported a prevalence of 55% for data of poor quality, which is lower than in our study, even though the criteria used were similar [15]. Likewise, the numbers reported in Mali and Senegal in the multi-center study performed in hospitals were lower than those in our study. In this case, the reason for the difference could be the fact that the study was performed after a project to improve the health information system in the locations studied [17]. In Tunisia in 2013, the prevalence of vaccination data of poor quality assessed at health facility level was lower than in our study, at 66%. The reason for this better performance in other countries could be the fact that the immunization programs in most of the countries are better monitored for data quality [18].

Our results confirm the role of behavioral factors in the framework of RHIS performance [4]. In our study, the health workers’ competence associated with their training, and their work engagement as a sign of motivation, were associated with the quality of the data. These findings support those of Hotchiss et al. in Uganda, which highlighted the relationship between behavior and RHIS performance, particularly regarding the use of the information [15]. In our study, we may consider perceived self-efficacy associated with the quality of the data in spite of the p-value which was borderline significant. This result supports the relationship highlighted by Hotchiss et al. in 2010 between perceived self-efficacy and RHIS performance [15]. The level of the health worker’s responsibility was also associated with the quality of the data in that the health workers who were also the managers of the health centers were less likely to produce reports of poor quality. This relationship could be considered paradoxical; the manager of the health center has a lot of other tasks and might devote less attention to data collection, while the health worker who is not responsible for the health center has more time to dedicate to RHIS tasks. On the other hand, as conveyed in the comments from the focus groups: “*When you are manager*, *you take care to avoid errors in your report because you do not want the medical officer to criticise your work in front of your colleagues at the monthly meeting*”. It is also clear that being the manager of the health center gives the health worker responsibility for the results submitted by the center (including the quality of the data produced), while the health worker who is not responsible for the health center is not under such pressure.

The sector of employment was associated with the quality of data in our study. This phenomenon may be explained in this context by the fact that the private sector in Benin is not significantly involved in the RHIS. Support activities such as training and supervision are, therefore, mostly dedicated to the public sector. Moreover, our findings confirm this difference of resources for the RHIS with the difference observed in our sample regarding supervision and availability of material resources for RHIS. Moreover, it is also interesting to note that despite the fact that the health workers had mentioned unsuitable RHIS training, training and retraining during the previous twelve months could have a positive impact on the quality of data. Improving the quality of training will produce improved results, particularly by tailoring it according to the following three components: the trainer, the health worker being trained and the tool being taught.

Although in our study organizational factors such as the availability of resources, supervision, financial incentives and the perceived complexity of the technical factors were not associated with the quality of the data, from a statistical point of view, the results of the focus group illustrate their role in data quality. This phenomenon is confirmed by the positive relationship found with the training. In the framework of RHIS performance, these factors were more directly linked with behavioral factors [4]; nevertheless, some authors have shown a direct positive link between financial incentives and performance [19,20]. Although this positive relationship between financial incentive and performance is still under investigation [20], with the experience of results-based financing in many developing countries, including Benin [20-22], it would be interesting to look at the issue in greater depth. We could accomplish this goal by incorporating, as a contractual performance result, an indicator for the quality of the data produced by the health center. In light of comments made by health workers in the focus groups, the format of the reporting form (design, number of items to fill, etc.) may need to be addressed. Shorter forms with pertinent items from the health workers’ point of view should certainly improve data quality. The main issue would be involving the health workers in the design process because the choice of indicators and thus form items is dependent on national and partner priorities.

It would have been worthwhile to perform modelling with adjustments, but taking into account the very low staff numbers in some categories of our sample, we were unable to accomplish this; this is one of the main study limitations. The comparisons made with other studies take into account the methods used in those studies because differences in methods could justify the differences in the findings. The analysis methodology we used together with LQAS sampling and the survival analysis is worthwhile, but it has its limitations, particularly in this study, where we had low staff numbers for certain categories, which made analyses with adjustment impossible. The simplicity of the LQAS methodology offers the opportunity to reproduce the work with more flexible criteria for evaluating data quality. Moreover, by working with a larger sample, more in-depth analyses could be conducted.

This study identified some factors associated with the quality of the RHIS data. The type of factors identified, such as those linked with the human resources as work engagement, self-perceived efficacy, and organizational factors show that the strategies for data quality improvement must focus on human resources, perhaps more than other resources. Indeed, in our context of limited resources, the first steps taken to improve the performance of RHIS should focus on investments in material and financial resources. Moreover, in a practical way, for example at the operational level, the choice of the staff delegated to data collection could take into account the relationship we found between data quality and the responsibility of the health worker.

The last assessment of health system of Benin showed that the health information system and the data quality is an important challenge; the ministry of health took the option to strengthen this field of health system [6,22]. The donors support the country in this strategic orientation as World Health Organization with introduction of a module for data quality assessment in the annual survey of Services Availability Readiness Assessment in 2013 [23]. The main challenges remain an optimal exploitation of existing opportunities such as different consultation frameworks for an effective private sector integration, a consensus between stakeholders (government, donors, private sector) for indicators choice, harmonized and lightweight reporting forms in that context where there is a compulsory need of revision for the reporting forms to improve data quality.

## Conclusions

This exploratory study identified some organizational and behavioral factors associated with the quality of the data in the RHIS in Benin. The methods could be reproducible on a larger scale and could aid in the development of inputs for strategic decision-making in human resources development for data quality improvement.

## Competing interests

The authors state that they have no financial or competing interests.

## Author’s contribution

YG was the principal investigator of the study and oversaw all aspects of the study design and implementation. She also drafted the manuscript LTO and AK performed the literature search, created the models, and reviewed the manuscript. MM and MWD supervised the study design, data management and statistical analysis, and drafting of the manuscript. YC was a co-investigator of the study and provided extensive feedback on the survey instruments and drafts of the manuscript. All authors read and approved the final manuscript.
